# *Vibrio *chromosomes share common history

**DOI:** 10.1186/1471-2180-10-137

**Published:** 2010-05-10

**Authors:** Benjamin C Kirkup, LeeAnn Chang, Sarah Chang, Dirk Gevers, Martin F Polz

**Affiliations:** 1 Dept. of Civil and Environmental Engineering, 15 Vassar Street, Cambridge MA 02139, USA; 2 Infectious Diseases, Childrens' Hospital Boston, 200 Longwood Ave., Boston MA 02115, USA; 3 Microbial Sequencing Center, The Broad Institute of MIT and Harvard, 7 Cambridge Center, Cambridge MA 02142, USA

## Abstract

**Background:**

While most gamma proteobacteria have a single circular chromosome, *Vibrionales *have two circular chromosomes. Horizontal gene transfer is common among *Vibrios*, and in light of this genetic mobility, it is an open question to what extent the two chromosomes themselves share a common history since their formation.

**Results:**

Single copy genes from each chromosome (142 genes from chromosome I and 42 genes from chromosome II) were identified from 19 sequenced *Vibrionales *genomes and their phylogenetic comparison suggests consistent phylogenies for each chromosome. Additionally, study of the gene organization and phylogeny of the respective origins of replication confirmed the shared history.

**Conclusions:**

Thus, while elements within the chromosomes may have experienced significant genetic mobility, the backbones share a common history. This allows conclusions based on multilocus sequence analysis (MLSA) for one chromosome to be applied equally to both chromosomes.

## Background

In traditional dogma, bacteria have one chromosome and a number of smaller DNA entities, like plasmids, which are propagated across generations unlinked to the chromosome. However, when bacteria have two chromosomes, are they permanently paired or do these physical entities recombine frequently relative to genes on these chromosomes? Since 1998, it has been known that some gamma proteobacteria have two chromosomes [1-3]. This followed discoveries that various other proteobacteria, namely alpha proteobacteria [4,5] and beta proteobacteria [6], could have multiple chromosomes as well. An initial debate occurred over whether the second *Vibrio *chromosome was really a 'chromosome' or whether it was merely a 'megaplasmid' [3,7]. The arguments for considering the second replicon a chromosome centered on its considerable size, essential gene content [8] and consistent stoichiometry. We can now add to that a unique replication machinery [9,10] that operates independently but in a coordinated fashion [11] with synchronous termination and thus consistent stoichiometry [12,13]. It is now accepted that most, perhaps all, *Vibrionaceae *(including the genera *Vibrio *and *Photobacteria*) have two chromosomes [14].

Genome analysis of the *Vibrios *rapidly uncovers variation even among closely related strains. Not only do the genome sizes differ widely [15], but even among conserved genes, there is incongruity among the inferred phylogenies. This is the well-accepted signature of horizontal gene transfer and homologous recombination. Gene organization also differs among sequenced strains, indicating large-scale genetic mobility. Individual genes and entire operons may be mobile among *Vibrio *[16-20]. In particular, Chromosome II varies widely in size and organization [14,21]. Further, many *Vibrio *carry (and presumably exchange) plasmids. Though it may seem unusual to expect as large a quantity of DNA to be transferred as an entire chromosome, there is evidence that *Vibrio *have experienced a transfer on that magnitude even recently: The putative *V. vulnificus *hybridization leading to biotype 3 involves very large quantities of DNA being transferred among *V. vulnificus *strains to create a hybrid strain almost evenly split in contributions from biotypes 1 and 2 [22]. However, the hybridization event involves loci from both chromosomes being transferred and appears to have preserved their associations with those chromosomes. As such, it does not appear to have been an exchange of chromosomal partners, but it raises the possibility that chromosomal exchange may have been an evolutionary mechanism within the *Vibrionaceae*.

The function of a second chromosome, and of multi-chromosomality in general, has been the subject of speculation [2,14,23]. That many of the genes on the *Vibrio *Chromosome II have specific environmental functions has been noted, and the role of the second chromosome in habitat adaptation has been tested experimentally [23]. Xu *et al *demonstrated that when *V. cholera *was grown in an animal host (rabbit ileal loop) a general shift in gene expression favored up-regulation of genes on the second chromosome relative to the gene expression profiles in exponential growth *in vitro*. This experimental data paired with the gross similarities among the chromosome I from all sequenced *Vibrio *and the great diversity of chromosome II, suggests that the second chromosome represents a collection of accessory elements and might be mobilized wholesale leading to a complete shift in habitat or niche [2,14].

'*Vibrio *phylogenies' that are built using MLSA or single-copy conserved genes typically use genes located on chromosome I [15,24-34] with the exception of intra-specific typing schemes for pathogens [17,22]. This is a side-effect of choosing stable, conserved, essential, single copy genes. However, it provides little assurance of representing the history of the entire genome given that Chromosome II is excluded from the analyses.

Given the high degree of mobility *Vibrio *genetic elements are presumed to have, it is possible that the two chromosomes have distinct and conflicting histories. There are currently 9 completely assembled *Vibrio *genomes available in the public databases and over 30 partially sequenced genomes. We explored these genomes to construct phylogenies for each of the two chromosomes using three approaches. First, single copy genes from each chromosome were assembled en suite and a phylogeny for each chromosome was inferred from these concatenated sequences. Second, the organization and gene content at the origins of replication of each chromosome (OriI and OriII for chromosomes I and II, respectively) were studied. Third, the genes from near the two chromosomal origins of replication were studied and their phylogenies estimated individually.

## Results and Discussion

### Chromosome Phylogenies

**Figure 1:**
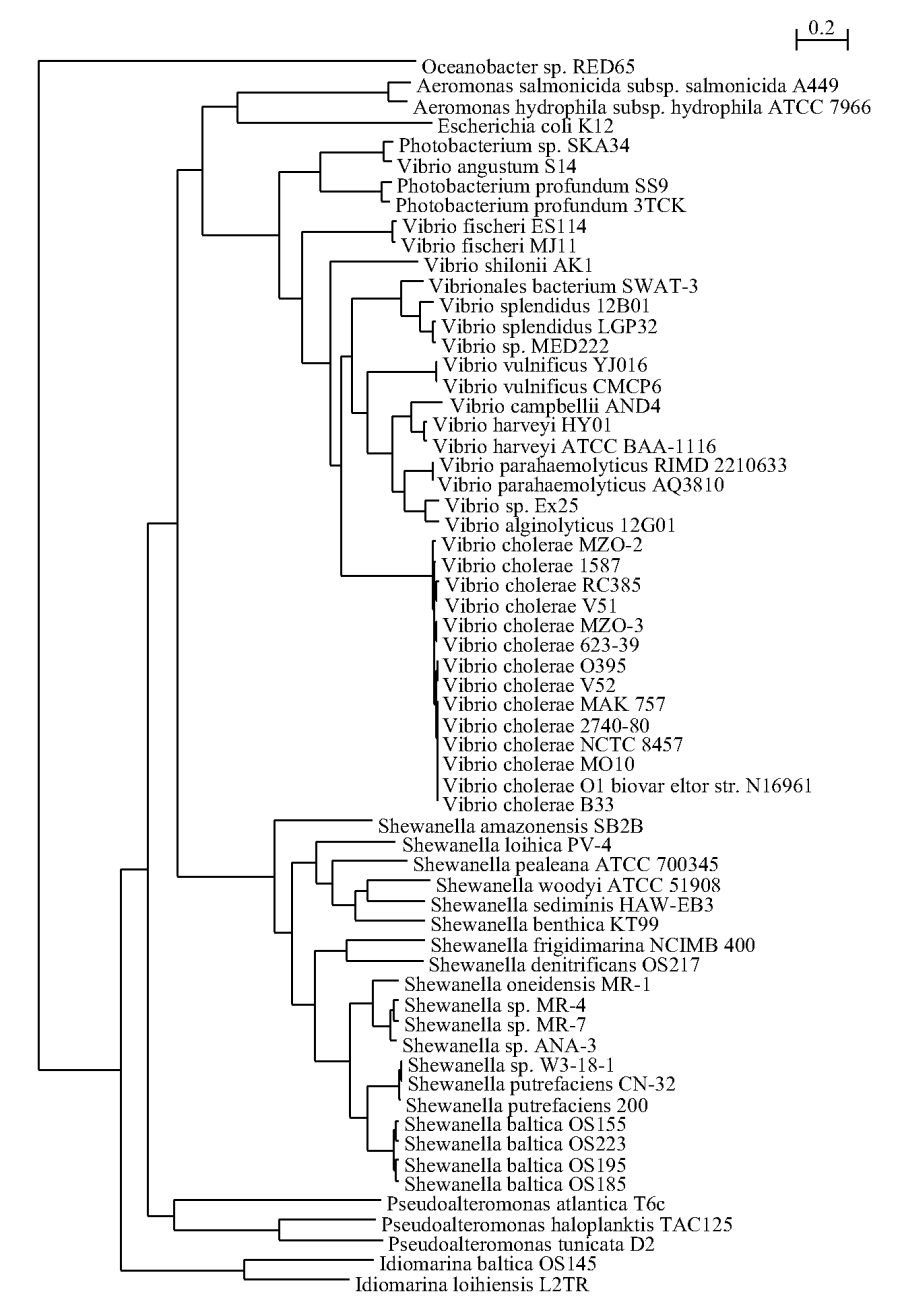
**Tree (Chromosome I)**. Inferred mean-field phylogeny of Chromosome I derived from a sampled concatenated gene sequence of single-copy orthologs distributed around the entire Chromosome I. The species tree is fully resolved and has 100% bootstrap support on all nodes outside of *V. cholerae *(1000 replicates). The list of genes and included locus tags is found in Additional file 1, supplementary materials.

**Figure 2:**
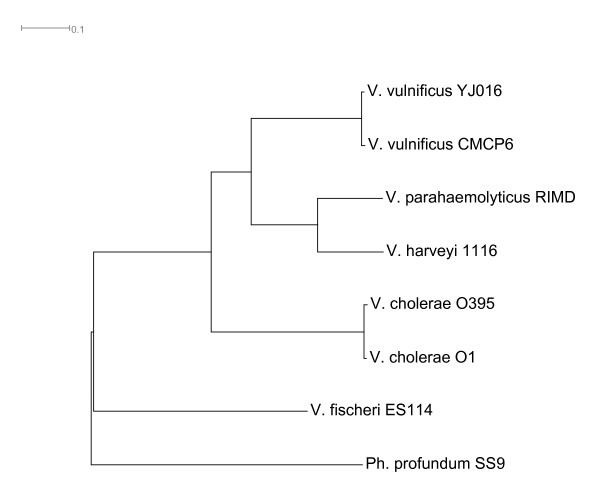
**Tree (Chromosome II)**. Inferred mean-field phylogeny of Chromosome II derived from a sampled concatenated gene sequence of single-copy orthologs distributed around the entire Chromosome II. The species tree is fully resolved and has 100% bootstrap support on all nodes (10000 replicates). The list of genes and included locus tags is found in Additional file 2, supplementary materials. Only closed genomes were included in this analysis.

The inferred phylogenies for the two chromosomes are congruent (Figures 1 and 2) and contain the expected major features, such as *Photobacterium *being basal to the *Vibrionaceae *and *V. fisheri *forming the next most basal clade. There are no unexpected sister taxa. The results of this analysis are compatible with published multi-locus analyses. However, instead of using 6 or 8 genes commonly used in MLSA, this analysis included 142 genes from chromosome I and 42 from chromosome II. These single copy genes include a range of functions including metabolism, information processing, flagellar structure and cytoskeletal components; as such, they represent sampling points from various pathways and genomic sections from around the entire genome. The concatenation of these well conserved genes provides a shared signal for the chromosomes as a whole, despite only composing a small fraction of the entire genome. The genes included in the analysis are listed under Additional files 1 and 2. The chromosome I tree is easily rooted by the various other genomes included in the analysis. All of these other clades fell together along accepted taxonomic lines. The most closely related strains in the tree are the *V. cholerae *strains; that clade is effectively unresolved because the internal distances are too short. The chromosome II tree cannot be rooted in the same manner as chromosome I because there is no obviously available outgroup: the chromosome II of *P. atlantica *is not homologous to the chromosome II of the *Vibrionaceae *being analyzed. However, rooting it identically by using the information from the chromosome I tree preserves the branching order of each tree. Thus, the 'mean field' approximation for the phylogeny of the two chromosomes is congruent at the species level. There is insufficient resolution among *V. cholerae *strains and too few members of other species to make inferences at a finer phylogenetic scale.

### Origin of Replication Organization

**Figure 3:**
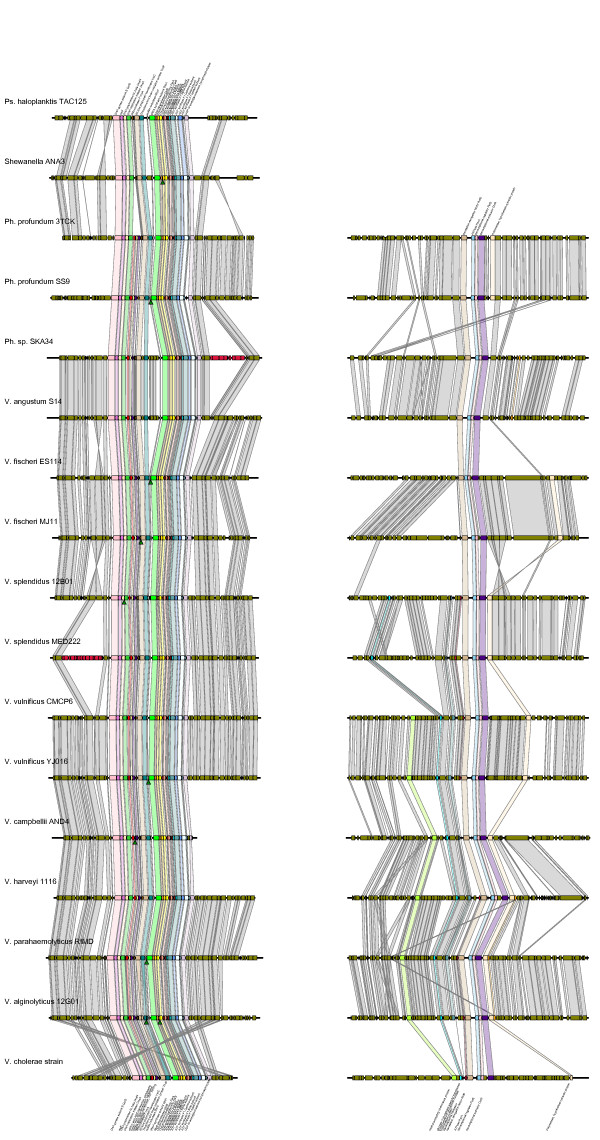
**OriI and OriII synteny figures**. The two origin regions of (A) Chromosome I and (B) Chromosome II. Open reading frames called in the annotated genomes are polygons pointing in the direction of their orientation. Colors label the open reading frames analyzed individually in estimating the phylogeny of the origin. The expanded figures with all labels are found in Additional files 3 and 4, supplementary materials.

The second method of analysis, studying the gene organization at the origins of replication (Ori), supported the finding that the two chromosomes share a single phylogeny at the species level. This method of analysis was more advantageously applied to chromosome II than chromosome I: Gene order in the region immediately surrounding the chromosome I origin appears too highly conserved between species to provide robust data on its phylogeny (Figure 3; expanded in Additional files 3 and 4). However, gene content is informative in that region suggesting that the species largely conform to the expected clustering even though the tree is not well supported (Figure 3). The difficulties are caused by a paucity of organizational changes that differentiate species at OriI - such as the inversion of three genes that sets apart the *V. fisheri*. Frequently, a change is unique to a sequenced strain and not shared by other members of its species. This can be extraordinarily disruptive of a distance estimate if the number of unique differences is large. In particular, at least three obvious saltations in the gene content introduce spikes of noise. In *V. cholerae *B33, an apparently mobile genetic region has imposed itself very close to the origin of replication. These 18 genes, almost as large as the region to be compared, interrupt an otherwise absolutely conserved region shared by the other *Vibrio cholerae*. A 9 gene region in *Photobacterium *sp. SKA34 contains several transposon and transposase genes. Similarly, 16 gene region in *Vibrio splendidus *MED222 interrupts an otherwise conserved region with a number of secretory system genes; it lacks apparent mobility elements which would explain its origin. Among the photobacteria, the flanking regions sometimes differ dramatically, as well, which disturbs the phylogeny with a very long branch, and the *Vibrio cholerae *appear to have inverted the entire region - but this would not impact a gene content analysis.

Gene presence/absence data is more informative in the regions surrounding the origin of chromosome II, even though across the breadth of the *Vibrionaceae*, the areas adjacent to the origin of replication have been relocated to such an extent that it is not practical to reconstruct their movement from as few genomes as are currently available. This is not unexpected, given how thoroughly shuffled chromosome II is relative to chromosome I [21]; see also Additional file 5 to explore the global rearrangement of chromosome II. Within a relatively short distance of the origin, however, genes can be reliably identified as orthologous and used in a presence/absence analysis. The origin was extended in each direction by 10 kb. As described in the methods, a gene presence/absence tree was constructed and this led to a distance tree entirely consistent with the mean-field approximation across Chromosomes I and II (i.e. Figures 1 and 2).

### Origin of Replication Genes

**Figure 4:**
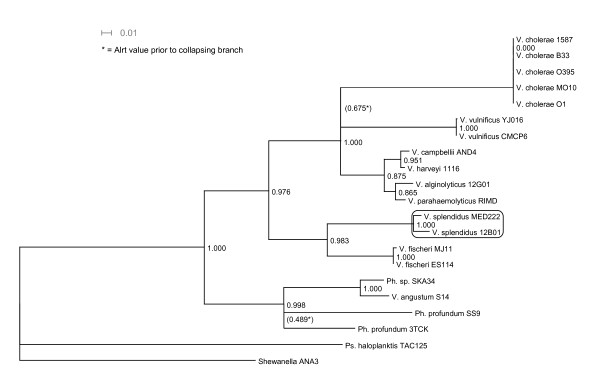
**Aberrant tree**. Tree inferred from the gene Asub on Chromosome I that is inconsistent with the trees inferred by other methods as described in this paper, including the trees for the individual gene phylogenies at other nearby genes. In this tree, the *V. splendidus *clade is found next to the *V. fisheri *clade, making it basal to its expected position. This tree is also referred to as "I" in Table 1, column 1. As shown, the tree is not fully resolved and branches with low support have been collapsed.

The phylogenies estimated for each of the gene families near the origin support the estimations derived from the two chromosomes overall. This third method of analysis led thus to the same conclusion as the other two. Table 1 lists the genes studied at each origin, focusing on their gene phylogeny, while Table 2 specifies the longer annotation names for the genes used in Table 1 and the type of data (DNA or AA) used to create the trees. The genes within the Ori regions are naturally subject to horizontal gene transfer and mutational noise, like all other genes. Two of them are too conserved or too noisy to present a clear phylogenetic signal over the *Vibrionales*. In these cases, ALrT (approximate likelihood ratio test) and bootstrap support are lacking across the entire tree (2/28 genes on chromosome I, 0 on chromosome II). Many other trees have limited support for individual clades. Clades with less than 0.05 ALrT [35] support or less than 70% bootstrap support were reduced to polytomies. In addition, the long branch of *V. cholerae *sometimes distorts other elements in the tree. In 8/28 trees from chromosome I and 2/12 trees derived from chromosome II, removing the cholera clade from the tree also restored a topology consistent with the mean-field tree in the other portions of the tree where previously it had been inconsistent with the hypothesis (labeled B in the first column of the table). Finally, one clade (*V. parahaemolyticus, V. alginolyticus, V. campbellii, V. harveyi*) was reliably monophyletic but presented numerous permutations in its internal structure. At OriI 9/28 genes presented diverse variants in this clade; at OriII, 3/12 genes presented variability within this clade. Ignoring this variation, 16/28 genes from chromosome I and 10/12 genes from chromosome II confirm the chromosomal phylogenies inferred by the above methods (labeled A). Finally, the remaining two genes on chromosome I lead to inferences that conflict with the others by placing *V. splendidus *in the *V. fischeri *clade (basal to its expected position, see Figure 4). Genes in OriI show more variation in phylogeny but fewer genes are available for study in OriII.

**Table 1:** Gene phylogenies for OriI and OriII.

	Relation to hypothesized chromosome phylogeny	Sequence set	Resolution	*V. alginolyticus/V. parahaemolyticus *clade
	**Consistent (A)** **Conditionally consistent (B)** **Uninformative (U)** **Inconsistent (I)**	**Complete(C)** **Incomplete (P)**	**Full (F)** **Partial (N)** **Unresolved (U)**	**Consistent with consensus(-)** **Scrambled (X)** **Other issues (see text) (O)** **Tree uninformative (n/a)**

**C1**				

LysR	A	P	N	-

MDR	A	P	F	-

UDP	I	C	N	-

Epsilon	B	C	N	O

Beta	B	C	N	O

Gamma	B	P	N	-

Alpha	A	C	N	-

Delta	A	C	N	X

Bsub	A	C	N	-

Csub	U	C	U	n/a

Asub	I	C	N	-

Isub	A	C	N	X

ParB	A	C	N	-

ParA	A	C	N	-

GidB	A	C	N	-

GidA	B	C	N	-

MioC	B	C	F	X

ThdF	B	C	N	X

YidC	A	C	N	X

RnpA	A	C	N	-

RpmH	U	P	U	n/a

ABC1	A	P	N	X

ABC2	A	P	N	-

ABC3	A	P	N	-

DnaA	A	C	N	-

DnaN	A	C	N	-

RecF	B	C	F	X

GyrB	B	C	F	-

**C2**				

MetC	A	P	F	X

GluP	B	P	N	X

PyrD	A	P	N	-

GTP	A	P	F	-

Hyp	A	P	N	-

TraR	A	P	N	-

RctB	A	C	N	-

ParA2	A	C	N	-

ParB2	A	C	N	-

ChrR	A	C	N	X

Poly	B	P	F	-

Chlor	A	P	F	-

All the genes analyzed are listed. The first column represents whether the estimated phylogeny was consistent with the hypothesized chromosome phylogeny (A and B, if removing cholera results in a consistent tree), inconsistent with the hypothesized phylogeny, or simply uninformative. The second column indicates whether all strains were represented for this locus and the third whether there were any clades outside the cholera clade at which the tree was a polytomy because of an uninformative or unsupported node. The fourth column includes cases in which *V. splendidus *is out of place in the tree (O) or where the *V. parahaemolyticus/V. alginolyticus *clade is not the same as in the consensus tree (X).

**Table 2:** Gene names.

Short name	Long name	Tree type
**C1**		

LysR	Transcriptional Regulator LysR	AA

Mdr	Multidrug Resistance Protein	AA

UPD	UDP-N-acetylglucosamine pyrophosphorylase	AA

Epsilon	ATP synthase F1 epsilon subunit	DNA

Beta	ATP synthase F1 Beta subunit	AA

Gamma	ATP synthase F1 Gamma subunit	AA

Alpha	ATP synthase F1 Alpha subunit	DNA

Delta	ATP synthase F1 Delta subunit	DNA

Bsub	ATP synthase F0 B subunit	DNA

Csub	ATP synthase F0 C subunit	n/a

Asub	ATP synthase F0 A subunit	DNA

Isub	ATP synthase F0 I subunit	DNA

ParB	Chromosome Partitioning Protein ParB	AA

ParA	Chromosome Partitioning Protein ParA	AA

GidB	Glucose inhibited division protein B	AA

GidA	Glucose inhibited division protein A	AA

MioC	Flavodoxin	AA

ThdF	Thiophene and furan oxidation protein	DNA

YidC	60 kDa inner membrane insertion protein	AA

RnpA	Ribonuclease P	AA

RpmH	Ribosomal protein L34	n/a

ABC1	Amino acid ABC transporter, ATP-binding protein	AA

ABC2	Amino acid ABC transporter, permease protein	AA

ABC3	Amino acid ABC transporter, periplasmic amino acid-binding portion	AA

DnaA	Chromosomal DNA replication initiator	AA

DnaN	DNA Polymerase III, beta chain	AA

RecF	Recombination Protein F	DNA

GyrB	DNA Gyrase	DNA

**C2**		

MetC	methyl-accepting chemotaxis protein	AA

GluP	glucose-1-phosphate adenylyl transferase	AA

PyrD	pyridoxamine 5'-phosphate oxidase	DNA

GTP	GTP cyclohydrolase II	DNA

Hyp	Hypothetical Protein	AA

TraR	transcriptional repressor	DNA

RctB	Putative Translation Elongation Factor	AA

ParA2	ATPases involved in Chromosome Partitioning	AA

ParB2	Predicted Transcriptional Regulator ParB	AA

ChrR	Transcriptional Activator ChrR	AA

Poly	Polymerase, hypothetical cytosolic protein	AA

Chlor	Chloramphenicol Acetyltransferase	AA

The genes surrounding the origins of replication otherwise identified by short names are described by their longer annotation names. In addition, the data (DNA or AA) used to create the trees is listed. This relates to the degree of conservation in the data; more conserved sequences require DNA trees to provide signal, less conserved sequences require AA trees to avoid excessive noise.

## Conclusions

Rampant horizontal gene transfer and plasmid exchange might create doubt as to the fidelity of paired chromosomes to one another. Further, this genetic mobility can create serious difficulties for anyone reconstructing a phylogeny for something as large as a chromosome, just as they do for someone inferring organismal and species phylogenies. Here, these difficulties have been overcome by using a range of methods that operate at different temporal and genetic scales. At the smallest scale, a number of individual gene phylogenies were reconstructed. At an intermediate scale, the gene content of a conserved region was used to infer a phylogeny. At the largest scale, concatenation of predominantly chromosome specific genes (though they may, in other genomes, be transferred among the chromosomes) provided an estimate of the history of the whole chromosome. In each case, the observed patterns were consistent - though, while many individual genes do not present a conflicting individual history, they may not support the hypothesis for lack of signal.

This congruence between the whole of the chromosome and the origin of replication suggests that the region around the origin of replication is either too large to relocate or is difficult to transfer because of its specific function. Individual genes in this region may experience horizontal gene transfer - witness the inclusion of a mobile genetic region in *V. cholerae *B33. Individual genes also appear amenable to transfer, deletion and insertion.

More than being able to create a relative history for each chromosome, it appears that since the origin of the two chromosomes in the ancestral *Vibrio*, they have continued as a pair. This suggests that they have also followed the cell itself; that we have a consistent phylogeny for the *Vibrio *species themselves - at least, the ones included in this analysis. Further genome sequencing would allow a similar analysis to provide the 'definitive' phylogeny of the *Vibrio*, but at much greater effort per strain than for MLSA [33]. MLSA schemes currently devised provide a mean field estimate of the phylogeny of Chromosome I; thus, as they are expanded to include increasing numbers of genes, those phylogenies are expected to agree with the phylogenies derived from studying the origins of replication. This suggests several genes that might be used in an MLSA of the *Vibrionaceae*, including Alpha, DnaN, and YidC from Chromosome 1 and ParA2 and GluP from Chromosome 2. These genes have potential primer sequences that are hypothetically capable of creating phylogenetic trees with the highest resolution and consistent signal so that they are comparable to the trees found in this study. It is a pleasing conclusion that separate MLSA schemes will not have to be executed for each chromosome independently.

## Methods

### Chromosome Phylogenies

Mean field approximation refers to the generalized phylogeny of the entire chromosome, regardless of differing histories. This was accomplished conceptually by means of concatenated gene trees for single copy homologous genes whose relatives are most easily determined and whose chromosomal affiliation is most certain. The restriction that the genes had to be single copy is meant to limit the analysis to orthologs while excluding paralogs. To select the genes for this analysis, a database of genomes was created. All the available *Vibrionaceae *(*Vibrio *and *Photobacterium*) genomes as well as an assortment of other gamma proteobacterial genomes (Additional file 6) were selected for analysis. All 62 genomes were broken down into lists of ORFs, which were entered into a MySQL database with their DNA and protein sequences as well as other identifying data. The entire suite of protein sequences were BLASTed against each other and the resulting hits were processed with orthoMCL v 1.4 to identify protein families [36]. A significant parameter used in orthoMCL was an inflation value of 1.5. Genes representing single copy gene families on the different chromosomes were aligned [37], stripped of their gaps, concatenated, and 100 kb, chosen as individual random sites, was chosen as the input for PhyML [38].

Phylogenies for *Vibrio *and *Photobacterium *chromosome I and II were based on the complete and incomplete published genomes with *P. atlantica *and *Shewanella sp*. ANA3 serving as the outgroup. Initially, *Pseudoalteromonas haloplanktis *was proposed as an outgroup for the chromosome II phylogeny. *P. haloplanktis*, unlike other sequenced pseudoalteromonads, has a second chromosome. However, that chromosome appears to have a distinct, plasmid-like origin of replication and a GC-skew that indicates unidirectional replication [39]. It shares several genes with the *Vibrio *chromosome II, but it is unclear how, if at all, it might share a substantive phylogeny with the *Vibrio *chromosome II. It contains presumably essential housekeeping genes, despite its otherwise plasmid-like features and likely represents a second origin of multi-chromosomality within the gamma proteobacteria. As a result, though genes from *P. haloplanktis *chromosome I were used as an outgroup to Vibrionaceae chromosome I, genes from *P. haloplanktis *chromosome II were not included in any analysis of Vibrionaceae chromosome II.

Initially, only completed Vibrionaceae genomes were analyzed for phylogeny of chromosome II. The incomplete genomes were then added to the analysis; genes represented multiple times in these genomes were excluded from the analysis. Incomplete genomes of *Vibrio cholerae *B33, *Vibrio harveyi *HY01, *Vibrio cholera *MZO-2, and *Vibrio angustum *S14 were excluded from this tree because they appeared to be missing members of gene families shared by the other genomes, even quite closely related conspecific strains. Finally, all the selected genes were processed as above, under the assumption that in the incompletely sequenced strains, genes particular to chromosome II in the complete genomes remained on chromosome II. With significantly fewer taxa in chromosome II than chromosome I, comparison for phylogenetic congruence involved eliminating a given taxa from the comparison if it was missing from one of the trees, and only using taxa present in both trees.

### Origin of Replication Organization

The origins of replication were studied first in the complete genomes, where they are identifiable by GC skew, annotation, and common gene content and organization. In the incomplete genomes, orthologous regions were identified by both gene content and skew. When the expected gene families and gene order coincided with appropriate shifts in skew, the origin was identified. For unfinished genomes, the origin could not be used in this analysis if it was broken up over several small contigs, but when the entire region was readily assembled in an unmistakable fashion, those contigs were included in the analysis.

The gene families derived from the above database were used to identify orthologs. Four core genes present in virtually all the genomes immediately at the origin were identified and used to anchor the analysis. From their furthest start and stop codons, regions 10 kb (OriII) and 20 kb (OriI) stretching outward were defined. These distances were chosen to balance issues of signal and noise. Particularly for OriI, a shorter region was uninformative because there were too few differences in gene content. For both of the chromosomes, as the regions grew larger, genome rearrangements were encountered that would wash out any signal from similarities in gene content at the origins themselves.

The genes within the selected regions were labeled by family and this data was used to produce a list of genes present in each region. This data was used to produce a pairwise distance estimate.

Where:

A = the smaller number of labeled genes in either of the two regions (i.e. in genome 1 or 2)

B = the number of families shared by the two regions (i.e. in the 10 or 20 kb regions on both genomes)

These pairwise distances were used to construct a square matrix; neighbor.exe from PHYLIP [40] was used to construct a neighbor-joining tree (settings; 10000 jumbles, root, otherwise default).

#### Origin of Replication Genes

The genes surrounding the origins of replication were grouped into families by similarity and synteny as detailed above. The phylogenies of the genes were estimated using PhyML-aLRT (settings; AA or DNA depending on data set, otherwise default) and strict consensus trees were created from the phylogenies. The individual gene trees were annotated with the necessary rearrangements to fit a largely resolved consensus tree. PhyML-aLRT was employed due to its ability to rapidly calculate the likelihood gain of all branches, allowing those without sufficient signal to be collapsed. As such, the cholera clade in particular contains insufficient divergence to be accurately resolved based on these genes.

The consensus tree arrived at by consensing the individual gene phylogenies estimated from genes near the origins of replication was compared to the trees derived from the other two methods. Common tools used for sequence and tree visualization included Dendroscope [41], BioEdit [42], and Artemis [43].

## Authors' contributions

BCK conceived of the project, generated the methods and drafted the manuscript. LC performed the final version of the analysis for each section and participated in writing the manuscript. SC performed an initial version of the first two analyses. DG developed the database for the research and reviewed drafts of the manuscript. MFP contributed ongoing critical review of the research aims and methods, extensively reviewed and edited the manuscript. All authors have read and approved the final manuscript.

## Supplementary Material

Additional file 1 **Chromosome I core table**. A key for the core genes on Chromosome I and their related locus tags from GenBank. Click here for file

Additional file 2 **Chromosome II core table**. A key for the core genes on Chromosome II and their related locus tags from GenBank. Click here for file

Additional file 3 **OriI synteny figure**. An expanded figure for OriI. Click here for file

Additional file 4 **OriII synteny figure**. An expanded figure for OriII Click here for file

Additional file 5 **Colinearity of Chromosome II**. The regions of homology among strains on chromosome II are not generally conserved in order or direction. Click here for file

Additional file 6 **Strains included table**. All the genomes included in the manuscript are listed with their genome sizes. Click here for file
