# Supplementary figures and images for: Vibrio chromosomes share common history
Source: BMC Microbiol. 2010 May 10;10:137. doi: 10.1186/1471-2180-10-137 (PMC2875227; doi:10.1186/1471-2180-10-137)

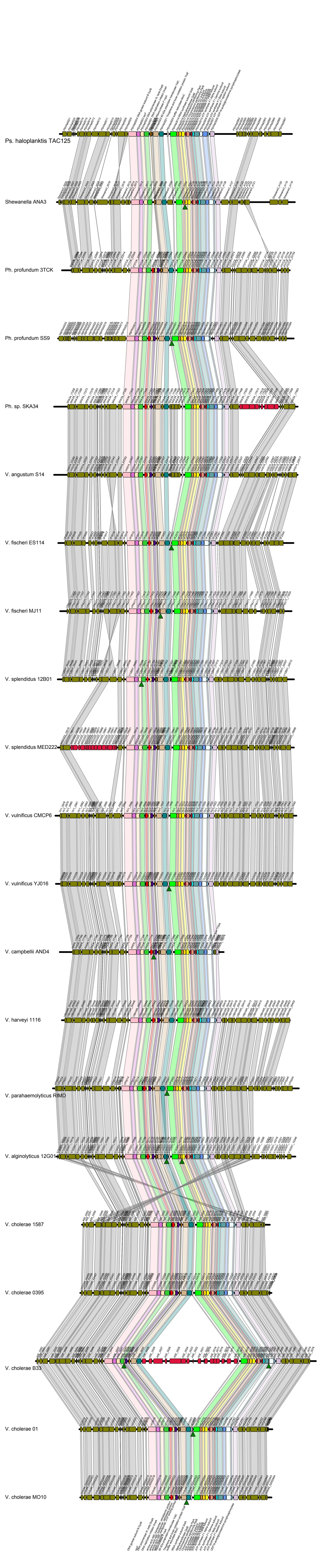

Supplement: Additional file 3 — OriI synteny figure. An expanded figure for OriI. [file 1471-2180-10-137-S3.PDF]
