# Supplementary material for: Vibrio chromosomes share common history
Source: BMC Microbiol. 2010 May 10;10:137. doi: 10.1186/1471-2180-10-137 (PMC2875227; doi:10.1186/1471-2180-10-137)
Supplement: Additional file 6 — Strains included table. All the genomes included in the manuscript are listed with their genome sizes. [file 1471-2180-10-137-S6.DOC]

| **Name** | **Chromosome 1** | **Chromosome 2** |
| --- | --- | --- |
| *Photobacterium profundum* 3TCK | 6,107,610 nt (unfinished) | |
| *Photobacterium profundum* SS9 | 4,085,304 nt | 2,237,943 nt |
| *Photobacterium* sp. SKA34 | 4,946,988 nt (unfinished) | |
| *Pseudoalteromonas haloplanktis* TAC125 | 3,214,944 nt | 635,328 nt (not included) |
| *Shewanella* sp. ANA-3 | 4,972,204 nt | n/a |
| *Vibrio alginolyticus* 12G01 | 5,160,431 nt (unfinished) | |
| *Vibrio angustum* S14 | 5,101,447 nt (unfinished) | |
| *Vibrio campbellii* AND4 | 4,255,798 nt (unfinished) | |
| *Vibrio cholerae* 1587 | 4,137,501 nt (unfinished) | |
| *Vibrio cholerae* 01 biovar eltor str. N16961 | 2,961,149 nt | 1,072,315 nt |
| *Vibrio cholerae* B33 | 4,026,835 nt (unfinished) | |
| *Vibrio cholerae* MO10 | 4,034,412 nt (unfinished) | |
| *Vibrio cholerae* O395 | 3,024,069 nt | 1,108,250 nt |
| *Vibrio fischeri* ES114 | 2,897,536 nt | 1,330,333 nt |
| *Vibrio fischeri* MJ11 | 2,905,029 nt | 1,418,848 nt |
| *Vibrio harveyi* ATCC BAA-1116 | 3,765,351 nt | 2,204,018 nt |
| *Vibrio parahaemolyticus* RIMD 2210633 | 3,288,558 nt | 1,877,212 nt |
| *Vibrio splendidus* 12B01 | 5,596,386 nt (unfinished) | |
| *Vibrio* sp. MED222 | 4,891,901 nt (unfinished) | |
| *Vibrio vulnificus* CMCP6 | 3,281,944 nt | 1,844,853 nt |
| *Vibrio vulnificus* YJ016 | 3,354,505 nt | 1,857,073 nt |
