# Supplementary material for: Vibrio chromosomes share common history
Source: BMC Microbiol. 2010 May 10;10:137. doi: 10.1186/1471-2180-10-137 (PMC2875227; doi:10.1186/1471-2180-10-137)
Supplement: Additional file 4 — OriII synteny figure. An expanded figure for OriII [file 1471-2180-10-137-S4.PDF]

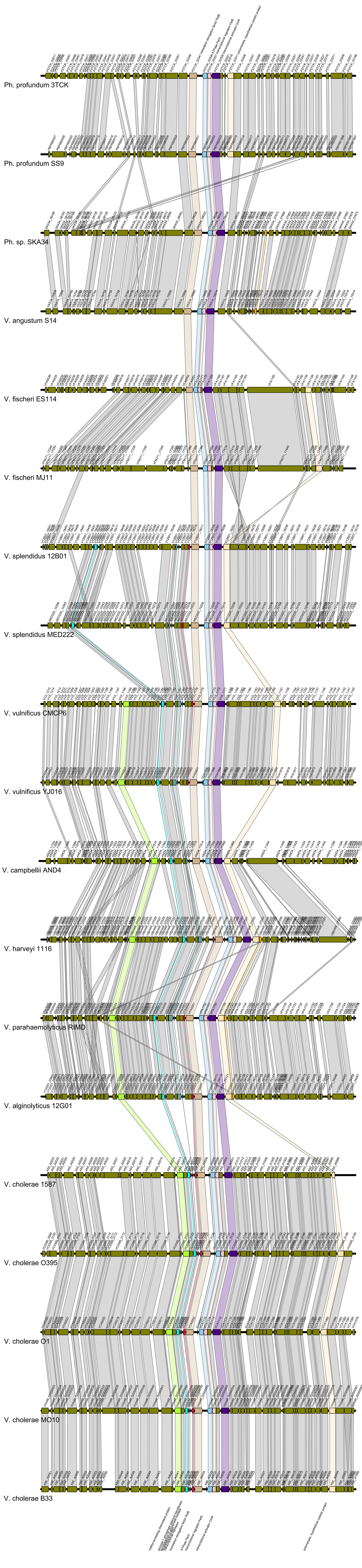

Ph. profundum 3TCK
Ph. profundum SS9
Ph. sp. SKA34
V. angustum S14
V. fischeri ES114
V. fischeri MJ11
V. splendidus 12B01
V. splendidus MED222
V. vulnificus CMCP6
V. vulnificus YJ016
V. campbellii AND4
V. harveyi 1116
V. parahaemolyticus RIMD
V. alginolyticus 12G01
V. cholerae 1587
V. cholerae O395
V. cholerae O1
V. cholerae MO10
V. cholerae B33
